# Transcriptome characterization and population genetics of *Ludisia discolor* (Ker Gawl.) A.Rich (Orchidaceae): implication for its conservation in Vietnam

**DOI:** 10.3897/BDJ.14.e173579

**Published:** 2026-01-08

**Authors:** Huan Trong Phan, Nguyen Thi-Lan, Oanh Kieu Le, Cuong Quoc Nguyen, Chung Thi Nhat Trinh, Yen Hieu Ha, Syed Noor Muhammad Shah, Duy Dinh Vu

**Affiliations:** 1 Coastal Branch of the Joint Vietnam-Russia Tropical Science and Technology Research Center, Khanh Hoa, Vietnam Coastal Branch of the Joint Vietnam-Russia Tropical Science and Technology Research Center Khanh Hoa Vietnam; 2 Hon Ba Nature Reserve, Khanh Hoa, Vietnam Hon Ba Nature Reserve Khanh Hoa Vietnam; 3 Fruit and vegetable research institute, Hanoi, Vietnam Fruit and vegetable research institute Hanoi Vietnam; 4 Vietnam National University of Agriculture, Hanoi, Vietnam Vietnam National University of Agriculture Hanoi Vietnam; 5 Department of Horticulture, Gomal University, Dera Ismail Khan, Pakistan Department of Horticulture, Gomal University Dera Ismail Khan Pakistan; 6 Joint Vietnam–Russia Tropical Science and Technology Research Center, Hanoi, Vietnam Joint Vietnam–Russia Tropical Science and Technology Research Center Hanoi Vietnam

**Keywords:** *Ludisia discolor*, Illumina HiSeq™ 4000, transcriptome, EST-SSR, genetic diversity, conservation

## Abstract

The combined pressures of overharvesting for ornamental and medicinal markets, along with pervasive habitat degradation, have precipitated a critical threat to wild populations of the jewel orchid, *Ludisia discolor*, within the biodiversity hotspots of Vietnam. The development of robust molecular tools is therefore essential to inform effective conservation strategies. This study established a suite of polymorphic Expressed Sequence Tag-derived Simple Sequence Repeat (EST-SSR) markers for *L. discolor* and applied them to assess genetic diversity and population structure. Transcriptome sequencing on the Illumina HiSeq™ 4000 platform yielded 44,764,702 high-quality reads. *De novo* assembly generated 26,271 unigenes (N50 = 2,160 bp; Q30 = 96.3%), which were comprehensively annotated against public databases (Nr, Swiss-Prot) and classified into Gene Ontology, KOG, and KEGG pathways. From 9,495 identified EST-SSR loci, 15 highly polymorphic markers were validated. Analysis of Vietnamese populations revealed moderate genetic diversity (Na = 3.17; He = 0.54; PIC = 0.59). Analysis of molecular variance (AMOVA) indicated significant genetic differentiation, with 23% of variation partitioned among populations. Bayesian clustering, Principal Coordinates Analysis (PCoA), and Neighbor-Joining (NJ) tree reconstruction consistently identified two distinct genetic clusters correlated with geography, suggesting restricted gene flow. These transcriptome-derived EST-SSR markers have proven effective for population genetic studies in *L. discolor*. The genomic resources and findings presented here provide a critical foundation for genetic monitoring and support the integration of molecular data into conservation and restoration programs for this ecologically and culturally significant orchid.

## Introduction

Orchids are globally threatened by a convergence of their unique biological constraints and intense anthropogenic pressures. Their often rare and fragmented populations, reliance on specific pollinators and mycorrhizal fungi, and complex life histories make them inherently vulnerable to extinction. This vulnerability is critically exacerbated by habitat loss, climate change, and illegal collection (Fay 2018). The scale of this crisis is starkly illustrated by their status under the Convention on International Trade in Endangered Species of Wild Fauna and Flora (CITES), where they account for more than 70% of all protected species (Ticktin et al. 2020).

**Figure 1.**
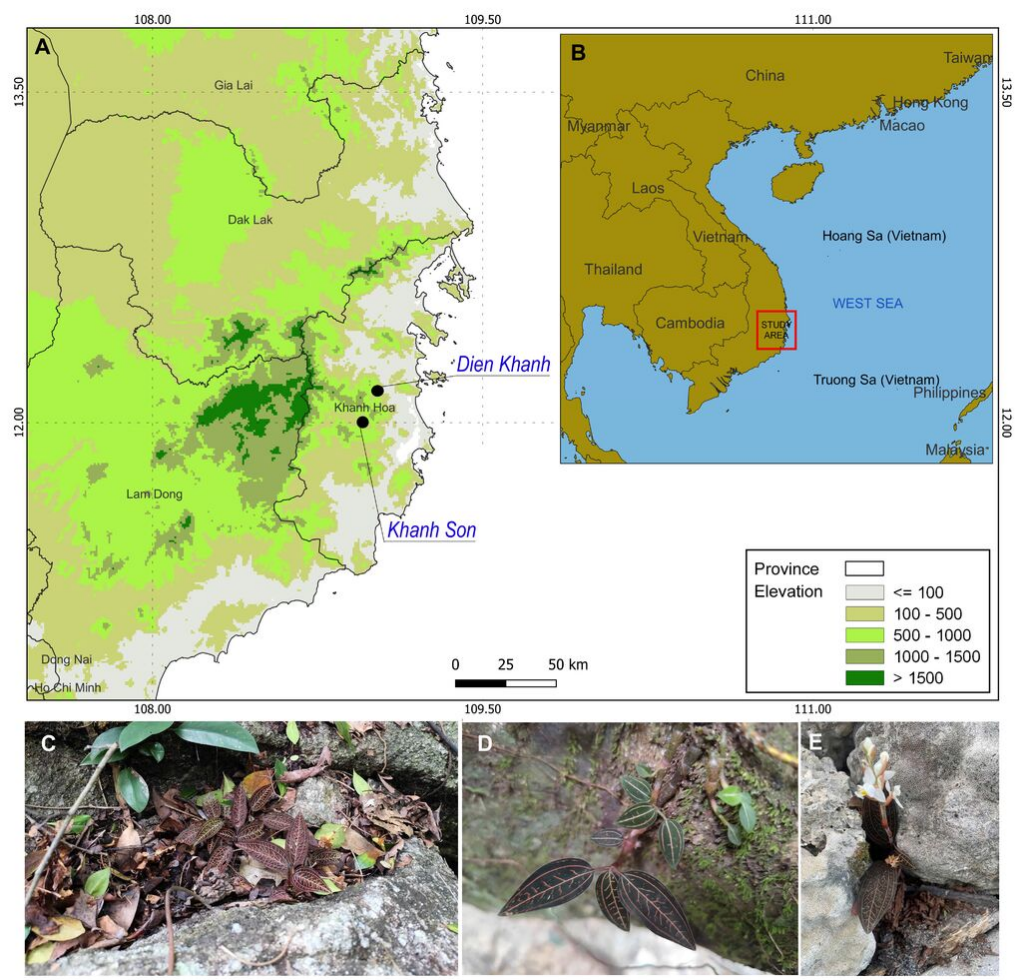
Map of field survey locations and geographic distributions of *L. discolor* Hon Ba Nature Reserve, Khanh Hoa Province, Vietnam. Map showing the collection sites (A and B), leaves (D), flowers (E) and adult plant (C)

*Ludisia discolor* (Ker Gawl.) A. Rich., the jewel orchid, is a terrestrial evergreen native to the shaded, humid forests of Southeast Asia. It spreads via succulent, creeping rhizomes that root at the nodes, sending up erect shoots reaching 15-25 cm in height. Leaves are alternate, ovate to elliptic; the adaxial surface is glossy, deep green to reddish-purple; the abaxial surface is purplish-pink. This coloration is consistent with adaptation to low-light understories. Inflorescences are terminal racemes, 3-8 cm long, bearing 1-12 white flowers. The perianth is galeate, formed by fused sepals and petals. The fruit is an ellipsoid capsule, 1.5 cm long and 4-5 mm wide, maturing from March to April (Fig. 1C, D, E) (Averyanov 2008, Averyanov et al. 2025). The species reproduces both sexually via seeds and asexually through vegetative fragmentation, demonstrating resilience in moist, foggy microhabitats (Krumov et al. 2022). *L. discolor* inhabits evergreen forest understories at 700-1500 m elevation across Southeast Asia and southern China (Averyanov et al. 2025). In Vietnam, it occurs singly or in small clumps on humus-rich soils or in rock crevices beneath dense canopies.

Beyond its horticultural value, *L. discolor* is widely used in traditional medicine. Phytochemical analyses have identified bioactive lactone glycosides – including kinsenoside, gastrodin, and goodyerosides A and B – which demonstrate hepatoprotective and anticancer activities (Righi et al. 2015, Yu et al. 2017, Poobathy et al. 2018). Kinsenoside demonstrates antitumor activity against lung cancer cell lines and exhibits hepatoprotective properties. A study in Vietnam reported that aqueous and ethanolic extracts (50-100 µg mL⁻¹) inhibited proliferation of HepG2 and A549 cancer cell lines by up to 15% (Nguyen et al. 2021). Despite this ecological and pharmacological importance, *L. discolor* remains genetically unexplored. Elevated conservation concern due to habitat loss, overharvesting, and degradation underscores a research bias toward chemistry and pharmacology, neglecting genomic and population genetic studies. Elucidating genetic diversity and population structure is critical for designing effective conservation strategies and understanding the genetic basis of ecological adaptation.

Over the past two decades, population genetics in Orchidaceae has been advanced by the application of various molecular markers (Luo et al. 2025, Tikendraa et al. 2021, Chung et al. 2013, Chung et al. 2012). Isozyme/allozyme analyses have elucidated population structure in *Pleurothallis*, *Bletilla*, and *Calanthe* (Borba et al. 2001, Chung et al. 2012, Chung et al. 2013). AFLP (Amplified Fragment Length Polymorphism) markers revealed genetic patterns in *Neotinea maculata*, *Liparis loeselii*, and *Orchis mascula* (Pillon et al. 2007, Duffy et al. 2008, Jacquemyn et al. 2009), while RAPD (Random Amplified Polymorphic DNA) and ISSR (Inter-Simple Sequence Repeats) markers quantified diversity in *Calanthe tsoongiana*, *Olea europaea*, and *Dendrobium chrysotoxum* (Martins- Lopes et al. 2007, Qian et al. 2013, Tikendraa et al. 2021). Among the molecular markers, SSRs (Simple Sequence Repeats, i.e., microsatellites) are especially valued for their codominance, high polymorphism, and reproducibility, establishing them as fundamental tools for population and evolutionary genetics (Varshney et al. 2005, Emanuelli et al. 2013, Ren et al. 2014, Vu et al. 2020, Yan et al. 2024). Traditional SSR development is laborious and expensive. Next-generation sequencing (NGS), particularly RNA-seq, has revolutionized marker discovery by enabling the efficient development of expressed sequence tag-derived SSRs (EST-SSRs) from coding regions (Jain 2011, Zalapa et al. 2011). EST-SSRs are cost-effective, transferable across related taxa, and functionally interpretable due to their origin in transcribed genes, making them valuable for studying adaptive and ecological traits (Ellis and Burke 2007, Yu and Li 2008, Tsai et al. 2016, Xie et al. 2020, Bai et al. 2023). While transcriptome sequencing has facilitated such studies in several non-model orchids (Wang et al. 2020, Bai et al. 2023, Jiang et al. 2024, Guan et al. 2025), no genomic resources currently exist for *L. discolor*. Consequently, the genetic basis of its ecological adaptation, stress responses, and population structure remains uncharacterized.

We generated foundational genomic resources for the orchid *L. discolor* through de novo transcriptome sequencing (Illumina HiSeq™ 4000). This study provides (i) a functionally annotated gene expression profile, (ii) a novel panel of validated EST-SSR markers, and (iii) an assessment of genetic diversity and population structure across wild Vietnamese populations. These resources establish a critical baseline for future genomic research, support conservation strategies for threatened populations, and offer insights into the adaptive evolution of this ecologically and medicinally significant species.

The long-term persistence of plant species is fundamentally linked to the maintenance of their genetic diversity. For the orchid *L. discolor*, its outcrossing breeding system and wind-dispersed seeds are undercut by ecological specializations and anthropogenic pressure. Dependence on specific pollinators and mycorrhizal symbionts, coupled with small, discontinuous populations, severely restricts realized gene flow and recruitment. This combination of traits predicts a genetic architecture vulnerable to erosion and divergence. Accordingly, we test two central hypotheses: first, that Vietnamese *L. discolor* populations retain only moderate to low genetic diversity, unlikely to achieve the high heterozygosity of robust outcrossing populations; and second, that their genetic variation is strongly partitioned into geography-linked clusters due to limited gene flow under ongoing habitat disturbance. To evaluate these predictions, we first generate foundational genomic resources through de novo transcriptome sequencing. We then apply newly developed EST-SSR markers to conduct a population genetic assessment, providing critical data to guide the conservation and management of this species in Vietnam.

## Materials and Methods

### Sample collection and RNA extraction

Fresh leaves, stems, and roots were collected from ten wild individuals of *L. discolor* at Hon Ba Nature Reserve, Khanh Hoa Province, Vietnam, in February 2025 (Fig. 1). The study received permission for research collaboration and the sample collection from Hon Ba Nature Reserve, Khanh Hoa Province, Vietnam (Admission number: 76/GGT-BQL date Feb 7, 2025). All samples were immediately flash-frozen in liquid nitrogen *in situ* and transported on dry ice to the Laboratory of Molecular Biology at the Joint Vietnam-Russia Tropical Science and Technology Research Center for storage at –80°C till RNA extraction. Total RNA was isolated using the Plant RNA Kit (Omega Bio-Tek, USA) with on-column DNase I treatment, following the manufacturer’s protocol. RNA integrity was assessed by 1% agarose gel electrophoresis and an Agilent 2100 Bioanalyzer (Agilent Technologies, USA); concentrations were determined with a NanoDrop ND-2000 spectrophotometer (Thermo Fisher Scientific, USA). Equal quantities of high-quality RNA from leaves, stems, and roots were pooled to construct a composite transcriptome library for sequencing.

### Illumina sequencing and de novo assembly

Poly(A)+ mRNA libraries were sequenced on the Illumina HiSeq™ 4000 platform (Illumina, USA) at Breeding Biotechnologies Co., Ltd. Raw reads were filtered to remove adapters, reads with >5% ambiguous bases (N), and reads with >20% of bases possessing a quality score (Q) < 10 using Trimmomatic v0.39 (Bolger et al. 2014). High-quality reads were assembled *de novo* using Trinity (Grabherr et al. 2011). The resulting contigs were clustered into non-redundant unigenes using TGICL v2.1 (Pertea et al. 2003).

### Functional annotation

Unigenes were annotated via BLASTX (E-value ≤ 1E–5) against the NCBI non-redundant protein (Nr) database (Deng et al. 2006), Swiss-Prot (Apweiler et al. 2004), Gene Ontology (GO) (Ashburner et al. 2000), Clusters of Eukaryotic Orthologous Groups (KOG) (Koonin et al. 2004), and Kyoto Encyclopaedia of Genes and Genomes (KEGG) (Kanehisa et al. 2004). Protein domains were identified with HMMER against Pfam (Mistry et al. 2021). GO terms were mapped and summarized with BLAST2GO (Conesa et al. 2005), and KEGG pathway analyses were used to infer putative biological functions.

### Development of EST-SSR markers

Microsatellites were identified from unigenes (>1 kb) using MISA (Beier et al. 2017). Detection thresholds were mononucleotides ≥12 repeats, dinucleotides ≥6, and tri- to hexanucleotides ≥5. A total of 6,437 EST-SSR primer pairs were designed in Primer3 v3.0 (Kõressaar et al. 2018) with the following criteria: primer length 18-24 bp (optimum 20 bp), annealing temperature 55-65°C (optimum 60°C), GC content 40-65% (optimum 50%), and expected amplicon size 100-300 bp. Selected primers were synthesized by Breeding Biotechnologies Co., Ltd.

### DNA extraction and microsatellite genotyping

For population genetic analyses, 68 individuals of *L. discolor* were sampled from two natural populations within Hon Ba Nature Reserve: Dien Khanh (DK, n = 24) and Khanh Son (KS, n = 44) (Fig. 1; Table 1). According to the manufacturer's instructions, genomic DNA was extracted from fresh leaves using the Plant DNA Kit (BioTeke, Beijing, China). DNA purity and concentration were assessed spectrophotometrically (NanoDrop ND-2000) and normalized to 20 ng µL⁻¹. A subset of 120 loci was selected for validation across 10 individuals from two populations. PCRs were performed in a 25 µL volume containing 2.5 µL template DNA, 12.5 µL 2× Taq Master Mix, 1 µL of each primer, and 8 µL nuclease-free water. Amplification was conducted on a GeneAmp PCR System 9700 (Applied Biosystems, USA) using the following protocol: initial denaturation at 94°C for 3 min; 40 cycles of 94°C for 30 s, primer-specific annealing for 30 s, and 70°C for 1 min; followed by a final extension at 72°C for 10 min. PCR products were sized and quantified using an Agilent 5300 Fragment Analyzer and the DNF-905 dsDNA kit (Agilent, USA).

### Genetic diversity analyses

Genotyping errors and null alleles were screened using Micro-Checker v2.0 (Van Oosterhout et al. 2004). Genetic diversity parameters: number of alleles (Na), effective number of alleles (Ne), observed heterozygosity (Ho), expected heterozygosity (He), inbreeding coefficient (Fis), and gene flow (Nm) were estimated with GenAlEx v6.5 (Peakall and Smouse 2012). Polymorphism information content (PIC) and deviations from Hardy-Weinberg equilibrium (HWE; 1,000 permutations) were assessed using Cervus (Kalinowski et al. 2007).

### Population structure and phylogenetic inference

Principal coordinate analysis (PCoA) was performed in GenAlEx v6.5 (Peakall and Smouse 2012). Population differentiation was assessed with pairwise Fst and hierarchical analysis of molecular variance (AMOVA; 10,000 permutations) in Arlequin v3.0 (Excoffier et al. 2007). Individual relationships were inferred via neighbor-joining (NJ) trees in Ntsys v2.2 (Rohlf 2005) and Mega v11.0 (Tamura et al. 2021). Bayesian clustering was implemented in Structure v2.3.4 (Pritchard et al. 2000) using an admixture model with correlated allele frequencies. For each value of *K* (1–15), ten runs were performed (burn-in: 100,000; MCMC: 500,000). The optimal *K* was determined using the ΔK method in Structure Harvester (Earl and vonHoldt 2011), with replicate runs aligned in Clumpp v1.1.2 (Jakobsson and Rosenberg 2007) and visualized in Distruct v1.1 (Rosenberg 2004).

## Results

### Illumina sequencing and de novo assembly

RNA-seq of *L. discolor* produced 44,764,702 high-quality clean reads (6.71 Gb) from 45,151,594 raw reads, with a sequencing error rate of 0.02%, a Q20 of 99.09%, a Q30 of 96.30%, and a GC content of 46.09%. *De novo* assembly generated 49,455 transcripts (total length 67,449,327 bp; mean length 1,364 bp; N50 = 2,034 bp). Following redundancy reduction, 26,271 unigenes were obtained (total length 44,915,839 bp; mean length 1,710 bp; N50 = 2,160 bp) (Table 2), demonstrating improved contiguity and reduced fragmentation. Length distribution confirmed efficient assembly: sequences of 201–300 bp decreased from 6,970 (transcripts) to 21 (unigenes), while long sequences (>2,000 bp) constituted 7,811 unigenes (29.7%). The resulting transcriptome provides a high-quality resource for functional annotation and marker development.

### Functional annotation and classification of unigenes

**Figure 2.**
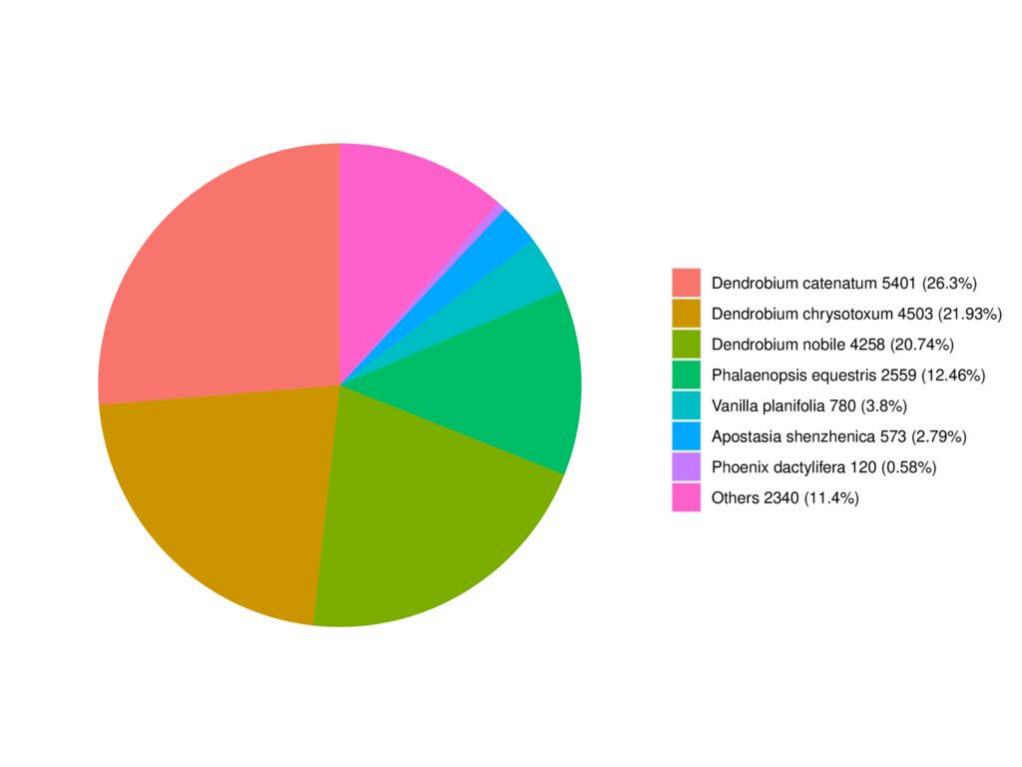
Distribution of species search of unigenes against the Nr database of *L. discolor*

**Figure 3.**
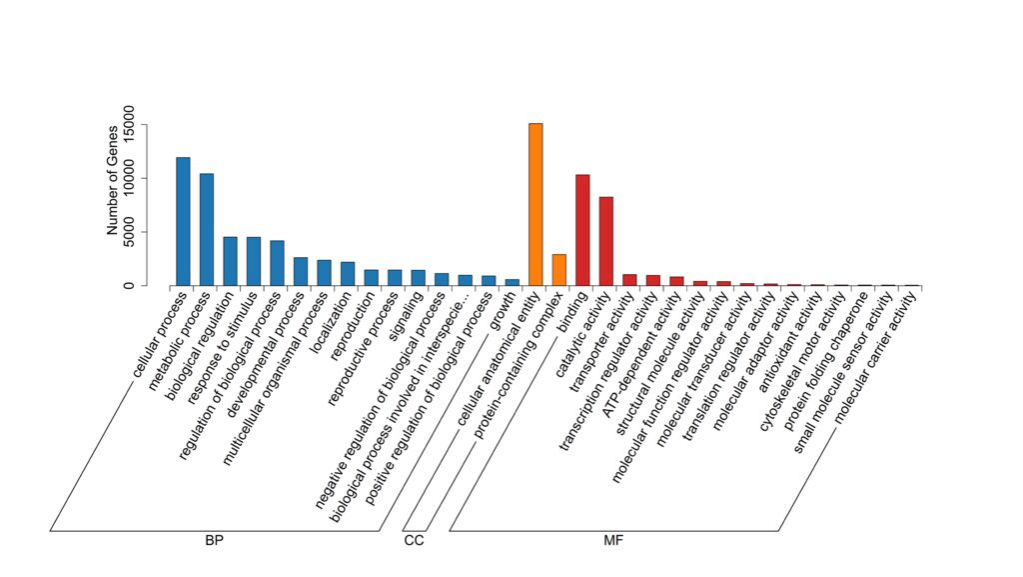
Gene Ontology (GO) classification of unigenes in transcriptome for *L. discolor*. Blue bars are associated with biological processes (BP); orange bars represent cellular components (CC); and red bars are linked to molecular functions (MF).

**Figure 4.**
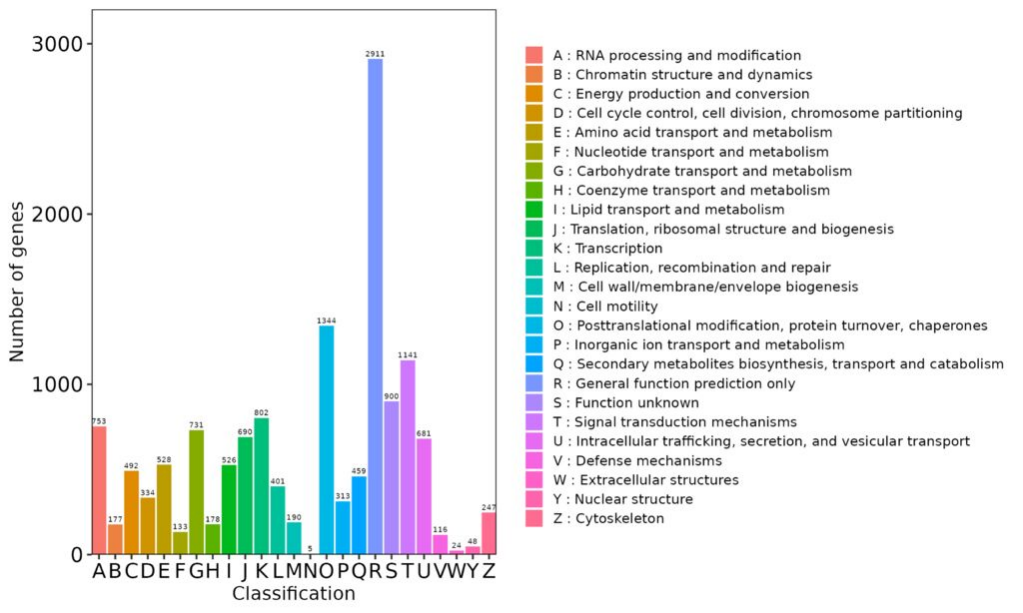
KOG functional annotation distribution of unigenes in transcriptome for *L. discolor*.

**Figure 5.**
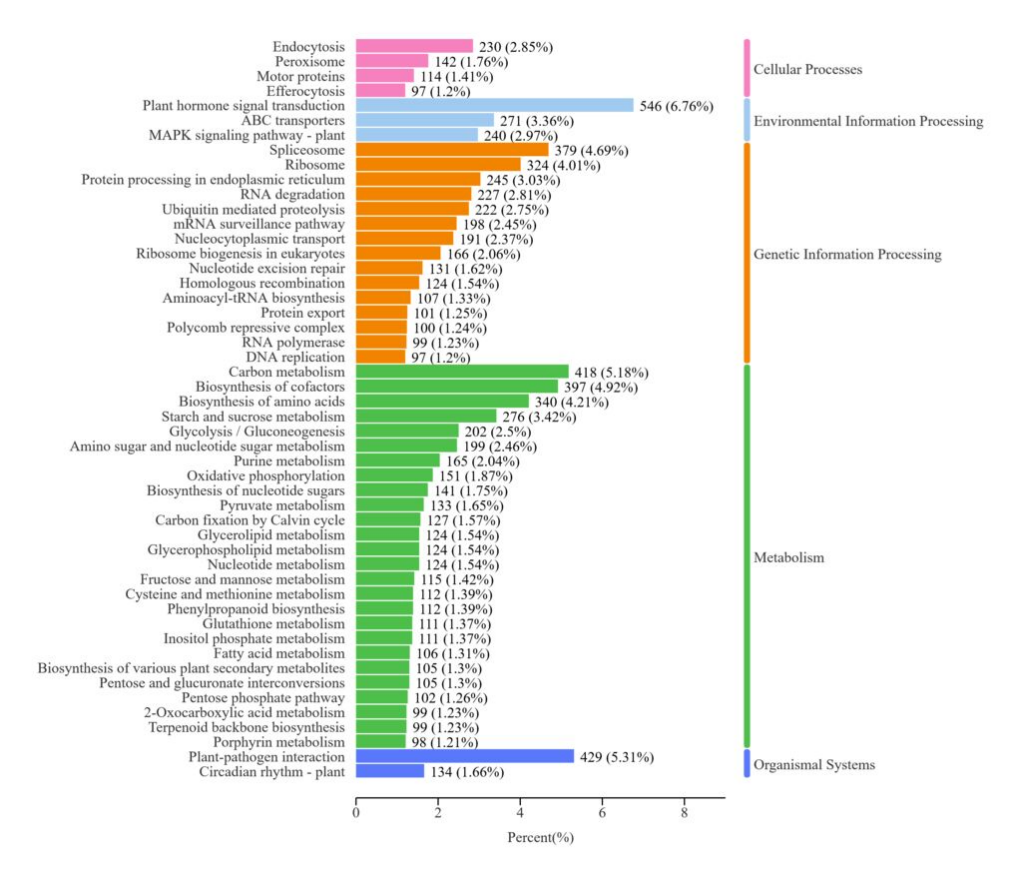
KEGG classification of *L. discolor* unigene.

Of the 26,271 unigenes, 20,644 (78.6%) were annotated in at least one database (Table 3). The highest annotation rates were against Nr (20,534; 78.2%) and TrEMBL (20,446; 77.8%), followed by GO (17,901; 68.1%), Pfam (16,131; 61.1%), KEGG (15,917; 60.6%), Swiss-Prot (15,361; 58.5%), and KOG (12,669; 48.2%). BLAST analysis against the Nr database revealed the high sequence similarity to orchid species, particularly *Dendrobium catenatum* (26.3%), *D. chrysotoxum* (21.9%), and *D. nobile* (20.7%) (Fig. 2). Gene Ontology (GO) classification assigned 17,901 unigenes to functional categories (Fig. 3, Suppl. material 1). Within biological processes, the "cellular process" (11,926) and the "metabolic process" (10,416) were the most abundant. "Cellular anatomical entity" (15,088) was the predominant Cellular Component term. For Molecular Function, "binding" (10,313) and "catalytic activity" (8,249) were the major categories. KOG classification assigned 12,669 unigenes to 25 functional categories (Fig. 4, Suppl. material 2). The most abundant categories were "General function prediction only" (2,911), "Posttranslational modification, protein turnover, chaperones" (1,344), "Signal transduction mechanisms" (1,141), and "Transcription" (802). KEGG pathway analysis assigned 15,917 unigenes, with 3,677 (45.7%) mapped to metabolic pathways (Fig. 5). These were predominantly enriched in carbohydrate metabolism and secondary metabolite biosynthesis. Significant enrichment was also detected in protein processing, plant-pathogen interaction, and hormone and MAPK signaling pathways, indicating functions related to ecological adaptation.

### Frequency and distribution of SSRs in the unigenes

**Figure 6.**
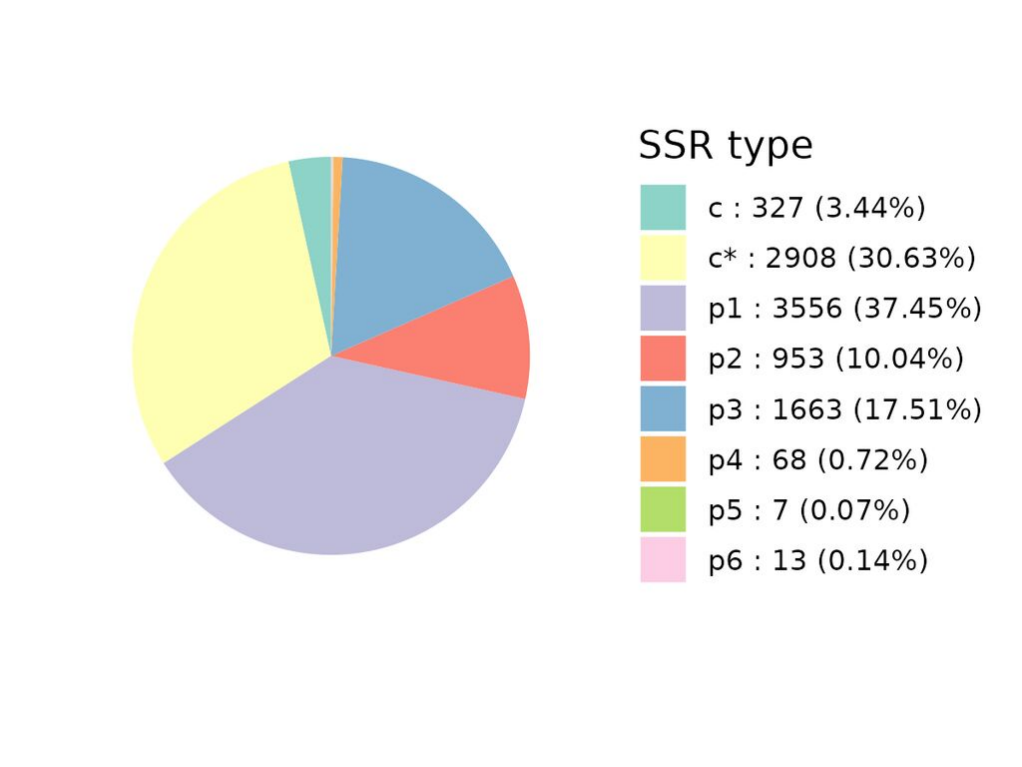
Distribution of different repeat type classes in *L. discolor* transcriptome.

A total of 9,495 EST-SSRs were identified from 26,271 unigenes (44.9 Mb assembly). SSRs were present in 7,597 unigenes (28.9%), of which 3,653 (48.1%) contained multiple SSRs, and 3,235 SSRs (34.1%) were in compound formation (Suppl. material 3). Motifs were categorized into eight classes (Fig. 6). Mononucleotide repeats were the most abundant (3,556; 37.5%), followed by compound (2,908; 30.6%) and trinucleotide (1,663; 17.5%) types. Dinucleotide repeats accounted for 10.0% (953), while tetra- (68; 0.7%), penta- (7; 0.07%), and hexanucleotide (13; 0.1%) repeats were rare. Trinucleotide motifs were predominant in coding regions, minimizing frameshift mutation risks. Analysis revealed a predominance of shorter simple sequence repeats (SSRs) (Table 4), with 5–6 repeat units being most frequent (34.3% and 29.7%) and those with ≥9 repeats being rare (<6%). Trinucleotide repeats (61.5%) were primarily composed of TCT, GAA, TGA, AAG, and CTT motifs. Dinucleotide repeats (35.2%) were dominated by CT, AG, TC, and GA (Suppl. material 4). This abundance of di- and trinucleotide SSRs in *L. discolor* represents a valuable resource for developing functional markers for population genetics.

### Genetic diversity

Of 6,437 designed EST-SSR primer pairs, 120 were screened across 10 individuals, yielding 44 polymorphic and 32 monomorphic loci. Fifteen robust polymorphic markers were selected for population-genetic analysis (Table 5). Population-level analyses revealed null alleles at three loci. Deviations from the Hardy-Weinberg equilibrium occurred at two loci in Dien Khanh (DK) and four in Khanh Son (KS). The mean number of alleles (Na) was 3.0 in DK and 3.33 in KS; the effective number of alleles (Ne) averaged 2.13 and 2.43, respectively. Observed heterozygosity (Ho) exceeded expected heterozygosity (He) in both populations (DK: Ho = 0.62, He = 0.51; KS: Ho = 0.63, He = 0.56) (Table 6). At the species level, mean values were Na = 3.17, Ne = 2.28, PIC = 0.59, Ho = 0.63, and He = 0.54. A negative mean Fis (−0.16) indicated overall heterozygote excess, though some loci showed localized deficiency. Population differentiation was moderate (Fst = 0.15), with substantial gene flow (Nm = 4.19) between the DK and KS populations.

### Genetic structure and cluster analysis

**Figure 7.**
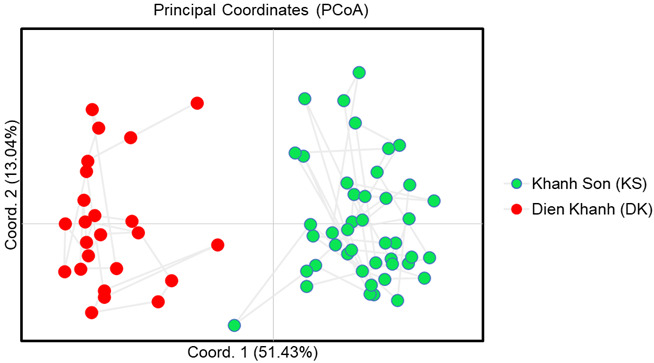
Principal Coordinates (PCoA) illustrating genetic relationship among different samples of *L. discolor* species.

**Figure 8.**
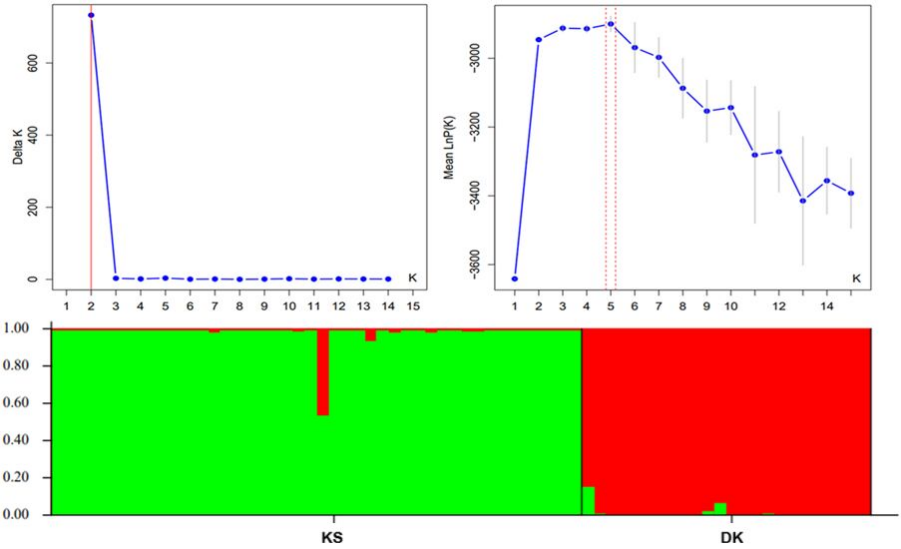
Genetic structure plot showing similarities and differences in the *L. discolor* populations K = 2 [From 1 to 44: Khanh Son (KS) in GREEN, and from 45 to 68: Dien Khanh (DK) in RED].

**Figure 9.**
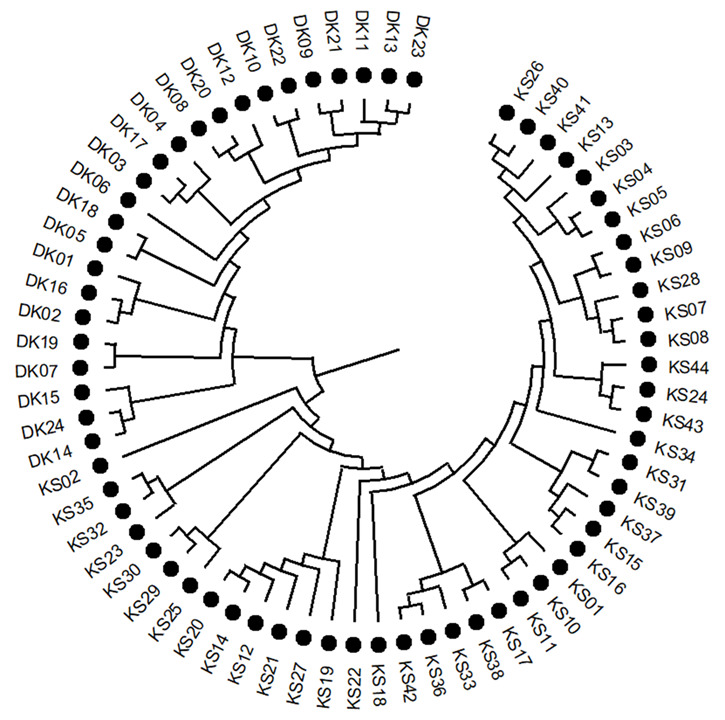
Neighbour-Joining (NJ) tree was constructed, based on Nei’s genetic distance of 68 genotypes of *L. discolor* based on 15 EST- SSR loci.

An analysis of molecular variance (AMOVA; 9,999 permutations) revealed that the majority of genetic variations (77%) occurred within individuals, with 23% partitioned among populations (Table 7). The overall population differentiation was significant (Fst = 0.255, *P* < 0.001). A negative Fis value (−0.141) indicated heterozygote excess, while the total inbreeding coefficient was low but significant (Fit = 0.150, *P* < 0.001). Principal coordinate analysis (PCoA) separated populations across the first three axes, which cumulatively explained 75.09% of the variance (Fig. 7). STRUCTURE analysis identified K=2 as the optimal number of genetic clusters (ΔK = 732.7), corresponding to the DK and KS populations (Fig. 8). The DK population formed a distinct cluster, whereas the KS population showed higher admixture, suggesting limited gene flow. A neighbor-joining tree based on Nei’s genetic distance also revealed clear separation between the two populations, with KS exhibiting greater internal substructure and DK forming a more homogeneous group (Fig. 9).

## Discussion

### Characterization of Transcriptome

We established the first transcriptomic resource for the endangered jewel orchid (*L. discolor)* through *de novo* RNA sequencing. The assembly yielded 26,271 unigenes with a mean length of 1,710 bp and an N50 of 2,163 bp, indicating high completeness. Functional annotation against seven databases (Nr, Nt, KEGG, KOG, Swiss-Prot, Pfam, GO) provided robust gene assignments for over 50% of the unigenes, creating a valuable genomic framework for future studies. Notably, comparative annotation profiling revealed significant differences between *L. discolor* and other Orchidaceae species. Only 26.3% of unigenes showed top homology to *Dendrobium catenatum* in the Nr database, indicating a substantial proportion of lineage-specific genetic elements. This divergence likely reflects distinct evolutionary paths driven by ecological specialization and reproductive strategies, consistent with the transcriptomic signatures of orchid diversification (Frech and Chen 2011, Chao et al. 2017, Jiang et al. 2018, Wang et al. 2020, Bai et al. 2023, Jiang et al. 2024, Guan et al. 2025). Similar patterns were observed in *Gastrodia elata*, where many unigenes lacked orthologs in related taxa, highlighting unique adaptive genetic features (Tsai et al. 2016, Wang et al. 2020). Our findings corroborate and extend the established evidence for substantial transcriptomic novelty in orchids, which appears intrinsically linked to their diverse life history and ecological interactions. Functional characterization of the *L. discolor* transcriptome confirmed its comprehensive biological scope, with unigenes assigned to 47 GO groups, 25 KOG categories, and 50 KEGG pathways covering core cellular and metabolic processes. Distinct from related species (*Cymbidium longibracteatum*, Jiang et al. 2018; *Phalaenopsis equestris*, Bai et al. 2023), the annotation revealed significant enrichment in stress response and secondary metabolic pathways, potentially reflecting adaptations to its understory habitat. This transcriptome serves as a critical genomic resource for *L. discolor*, enabling future research in gene discovery, evolutionary genomics, secondary metabolite biosynthesis, and the development of molecular markers for conservation genomics.

### Frequency and distribution of EST-SSRs

The mean unigene length assembled for *L. discolor* (1,710 bp) exceeded that of most previously characterized orchid transcriptomes, including *Gastrodia elata* (1,592 bp; Tsai et al. 2016), *Ophrys sphegodes* (1,178 bp; Sedeek et al. 2013), *Orchis italica* (1,556 bp; De Paolo et al. 2014), *Cypripedium formosanum* (1,679 bp; Chao et al. 2017), *Calanthe masuca* (1,196 bp), and *C. sinica* (1,086 bp; Hu et al. 2018). This greater average contig length is advantageous for Expressed Sequence Tag-Simple Sequence Repeat (EST-SSR) marker development, as it facilitates more efficient in silico identification of microsatellite loci and the design of flanking primers (Zalapa et al. 2011). Consistent with this, a total of 9,495 EST-SSRs were identified in the *L. discolor* transcriptome. This quantity is higher than that reported for related species such as *C. masuca* (3,764) and *C. sinica* (7,189) (Hu et al. 2018). The observed abundance of SSRs is likely attributable to the high quality of the de novo assembly, as well as to species-specific genomic characteristics that are known to influence microsatellite density (Wei et al. 2011). Analysis of SSR loci revealed a diverse array of repeat motifs. Mononucleotide repeats were the most prevalent (37.45%), followed by trinucleotide repeats (17.51%). This predominance of mono- and trinucleotide motifs is congruent with findings in other orchid transcriptomes (Chao et al. 2017, Hu et al. 2018), indicating a conserved structural organization of genic microsatellites within the Orchidaceae family. The prevalence of trinucleotide SSRs is of particular functional significance. Due to the triplet nature of the genetic code, expansions or contractions in these motifs are less likely to cause frameshift mutations compared to dinucleotide repeats (Li 2004). This suggests that a substantial proportion of the identified SSRs in *L. discolor* are situated within coding regions and may be associated with gene regulatory functions or adaptive processes. This observation is supported by studies in *Dendrobium* and *Phalaenopsis*, where an enrichment of trinucleotide repeats was documented in genes involved in stress response and secondary metabolism (Zhang et al. 2013, Bai et al. 2023). The high frequency and broad distribution of these EST-SSRs underscore their utility as molecular markers. A key advantage of EST-SSRs over genomic SSRs is their higher cross-species transferability (Kong et al. 2006), as they are derived from conserved expressed genes. Furthermore, their location within transcribed regions increases the probability of functional relevance, making them exceptionally valuable tools for applications in the conservation genetics of threatened orchid species.

### Genetic diversity and population structure

Genetic diversity, a fundamental determinant of evolutionary potential and long-term species viability (Li et al. 2021), is governed by biogeographic history, effective population size, and reproductive strategy (Gijbels et al. 2015). Consequently, widely distributed, outcrossing taxa typically exhibit elevated diversity (Novak 2020), which enhances adaptive capacity to environmental change (Höglund 2009). Empirical studies confirm high heterozygosity (Ho) in several *Cymbidium* species (e.g., *C. goeringii*, Ho=0.81) using EST-SSR markers (Liu et al. 2014, Ning et al. 2023). In contrast, *L. discolor* demonstrates moderate diversity (mean Ne=2.28, Ho=0.63, He=0.54), a pattern also observed in other orchid species like *C. ensifolium* and *Dendrobium officinale* (Li et al. 2015, Xie et al. 2020). This attenuation is likely attributable to historical biogeographic isolation compounded by anthropogenic pressures such as deforestation and overharvesting. A significant proportion of genetic variance is partitioned among populations, underscoring the conservation imperative of maintaining multiple populations to preserve species-level diversity. Population size reduction can strongly influence allelic richness and heterozygosity (Lowe et al. 2005, Leimu et al. 2006). Our analysis suggests habitat degradation and overexploitation are the primary causes of genetic decline in *L. discolor*. The Diên Khánh population exhibits markedly lower genetic diversity than the Khanh Son population. This reduction intensifies population isolation and the effects of genetic drift and inbreeding, thereby increasing extinction vulnerability (Honnay and Jacquemyn 2007, Bürli et al. 2025). Furthermore, the observed genetic variance and structure align with the influences of historical range, contemporary gene flow, population size, and reproductive biology (Yun et al. 2020). Genetic differentiation in the widespread outcrossing species *L. discolor* is moderate, consistent with patterns observed in other orchid species. An AMOVA revealed significant population structure (Fst = 0.255, P < 0.001), with 23% of genetic variation partitioned among populations. This level of differentiation is comparable to that reported for *Epipactis helleborine* (Fst = 0.206) and *Pelatantheria scolopendrifolia* (Fst = 0.302). Despite an estimated gene flow of Nm = 4.19, the significant Fst value suggests dispersal is sufficient to prevent fixation but insufficient to homogenize populations, a pattern characteristic of species with fragmented habitats. This aligns with the expectation that rare terrestrial orchids exhibit greater genetic structure than common congeners (Phillips et al. 2012). Despite wind-mediated seed dispersal, orchid recruitment is contingent on specific mycorrhizal fungi, thereby restricting realized gene flow (Soliva and Widmer 2003). Consequently, although genetic analyses (e.g., STRUCTURE, PCoA) reveal localized admixture, dispersal and recruitment limitations enforce significant population genetic structure (Alcantara et al. 2006).

### Conservation implications

Genetic diversity is fundamental for species persistence and effective conservation (Höglund 2009). Although *L. discolor* is native to a broad range in Southeast Asia and reproduces by outcrossing with wind-dispersed, dust-like seeds, Vietnamese populations often occur as small, elevationally discontinuous and spatially fragmented sub-populations, where realized gene flow is restricted by habitat disruption and recruitment constraints. Our results showing moderate genetic diversity and significant genetic structure among populations suggest continued vulnerability to genetic drift and anthropogenic genetic erosion.Within the sampled range, Khanh Son (KS) exhibits significantly higher allelic richness than Dien Khanh, identifying KS as an important genetic reservoir. Therefore, Hon Ba Nature Reserve should be prioritized for long-term population reinforcement, especially using genetically characterized material from KS for intra-reserve translocation, while preserving local genetic structure. Beyond Hon Ba, other protected evergreen forest areas in the south-central highlands, such as forest compartments in neighboring conservation zones with similar canopy shade, humidity, and elevation gradients, should be surveyed as candidate sites for future assisted translocation only after confirming habitat suitability and existing population status.To prevent overharvesting, we recommend: (i) strict enforcement of a ban on wild collection inside protected areas; (ii) expansion of ex situ propagation through tissue culture and certified nurseries to supply ornamental and medicinal markets with cultivated plants; and (iii) community-driven awareness and alternative livelihood programs in buffer-zone villages to shift all plant use from wild-sourced to nursery-grown material. The novel EST-SSR markers developed here provide an effective tool for long-term genetic monitoring, helping detect early allele loss, track diversity trends, and inform breeding and restoration programs.We emphasize that sustainable conservation of *L. discolor* in Vietnam requires an integrative framework combining genetic, ecological, and demographic evidence, implemented across multiple populations, monitored over time, and supported by habitat protection, cultivated plant supply chains, and community participation.

## Conclusions

The integration of molecular data is critical for effective conservation planning. Our development of transcriptomic resources and EST-SSR markers for *L. discolor* provides a foundation for long-term population monitoring, functional genomics, and active species management. Furthermore, the transferability of these functionally relevant markers to other threatened orchid taxa in Southeast Asia presents a strategic resource for regional conservation efforts aimed at preserving genetic diversity and adaptive capacity in the face of habitat loss and overexploitation.

## Supplementary Material

D05A8FFF-B7AA-53EC-AEAC-88F8378A1EBC 10.3897/BDJ.14.e173579.suppl1 Supplementary material 1 Gene Ontology Data type Table S1 Brief description Gene Ontology (GO) classification of unigenes in transcriptome for *L. discolor*. File: oo_1479670.docx https://binary.pensoft.net/file/1479670 Trong Huan Phan, Thi Lan Nguyen, Thi Kieu Oanh Le, Quoc Cuong Nguyen, Thi Nhat Chung Trinh, Hieu Yen Ha, Dinh Duy Vu

CF26F57D-9DD5-5C1A-A43B-054DB4500986 10.3897/BDJ.14.e173579.suppl2 Supplementary material 2 KOG functional annotation distribution Data type Table S2 Brief description KOG functional annotation distribution of unigenes in transcriptome for *L. discolor*. File: oo_1479671.docx https://binary.pensoft.net/file/1479671 Trong Huan Phan, Thi Lan Nguyen, Thi Kieu Oanh Le, Quoc Cuong Nguyen, Thi Nhat Chung Trinh, Hieu Yen Ha, Dinh Duy Vu

FE5347B0-C0C4-5F4B-BEF7-D7D1A975A886 10.3897/BDJ.14.e173579.suppl3 Supplementary material 3 Summary of analyses Data type Table S3 Brief description Summary of analyses of expressed sequence Tag–Simple Sequence repeat (EST-SSRs) in *L. discolor*. File: oo_1479673.docx https://binary.pensoft.net/file/1479673 Trong Huan Phan, Thi Lan Nguyen, Thi Kieu Oanh Le, Quoc Cuong Nguyen, Thi Nhat Chung Trinh, Hieu Yen Ha, Dinh Duy Vu

6C045B21-AB14-53F5-9CA6-CE185ED77D10 10.3897/BDJ.14.e173579.suppl4 Supplementary material 4 Summary of SSR types Data type Table S4 Brief description Summary of SSR types in the transcriptome of *L. discolor*. File: oo_1479674.docx https://binary.pensoft.net/file/1479674 Trong Huan Phan, Thi Lan Nguyen, Thi Kieu Oanh Le, Quoc Cuong Nguyen, Thi Nhat Chung Trinh, Hieu Yen Ha, Dinh Duy Vu

## Figures and Tables

**Table 1.** Collection of localities of *L. discolor* for SSR analysis.

**Populations**	**Collection locality**	**Altitude (meters)**	**Latitude (N) / Longitude (E)**		**Sample size**
Dien Khanh (DK)	Hon Ba Nature Reserve, Khanh Hoa Province	415	12°08'23.4'', 109°00'36.8''		24
Khanh Son (KS)	Hon Ba Nature Reserve, Khanh Hoa Province	608	12°02'54.7'', 108°58'32.5''		44

**Table 2.** Length distribution of assembly transcripts and unigenes for *L. discolor*.

**Length range (bp)**	**No. Transcript**	**No. Unigene**
201-300	6,970	21
301-400	3,953	256
401-500	2,959	1,006
501-600	2,373	1,647
601-700	2,115	1,714
701-800	1,953	1,511
801-900	1,764	1,297
901-1000	1,783	1,229
1001-1100	1,716	1,173
1101-1200	1,691	1,158
1201-1300	1,610	1,095
1301-1400	1,602	1,068
1401-1500	1,504	960
1501-1600	1,486	986
1601-1700	1,393	952
1701-1800	1,300	860
1801-1900	1,172	807
1901-2000	1,089	720
>2000	11,022	7,811
Total Number	49,455	26,271
Total Length	67,449,327	44,915,839
N50 Length	2,034	2,160
N90 Length	675	869
Mean Length	1,364	1,710

**Table 3.** Functional annotation of *L. discolor* in different databases.

**Annotated database**	**No. Annotated**	**Percentage (%)**
KEGG	15,917	60.59
Nr	20,534	78.16
SwissProt	15,361	58.47
TrEMBL	20,446	77.83
KOG	12,669	48.22
GO	17,901	68.14
Pfam	16,131	61.14
Annotated in at least one database	20,644	78.58
Total Unigenes	26,271	100

**Table 4.** Length distribution of SSRs based on the number of repeat units of *L. discolor*.

**Number of repeat units**	**Di**-	**Tri**-	**Tetra**-	**Penta**-	**Hexa**-	**Total**	**Percentage (%)**
5	-	869	47	5	6	927	34.28
6	394	396	9	1	4	804	29.73
7	268	262	3	1	2	536	19.82
8	158	117	8	0	0	283	10.47
9	133	19	0	0	1	153	5.66
10	0	0	0	0	0	0	0.00
>10	0	0	1	0	0	1	0.04

**Table 5.** Primer sequences, repeat motif, size range of alleles, and annealing temperature of 15 polymorphic EST-SSR markers developed for *L. discolor*.

**Locus**	**Sequence of primer (5'–3')**	**Motif type**	**Size (bp)**	**Ta (°C)**
LG01	F: ATCTGGATGGAGCGCTCTCT R: CTGCTCCGATGATCACCACT	(CTCG)5	116-130	56
LG02	F: GCTAGTGCATTTGCTGTGGG R: GCAGAGGCATGCTCAACAAC	(TTG)9	224-257	55
LG03	F: TTCCCCACAGAGAAAGCAGC R: GGGTTGCAGCTTCAGTACCT	(GACC)5	242-262	54
LG04	F: GGGAATTCCTGGCATAAAGAGC R: CTAACAAGCCCTGCTTCCCA	(AG)9	242-281	55
LG05	F: TGTGCTTGCTCTGACTTCTGA R: CTGGAGAGGAGACCAACTGC	(AT)9	154-182	55
LG06	F: CCACAATGTGAGAAAAGTCCCA R: CTGTAGTGGAGGCAGCAACA	(TTTC)5	260-296	55
LG07	F: GTTGGGACTGATGATGGCCA R: AGCAGGCTAGGCTGTTTACA	(TTTA)5	196-216	53
LG08	F: AGTTTGGACCATTGGGCACT R: GGACAGGTCCTCTCTGCAAC	(AATC)6	112-116	55
LG09	F: GGCCGTTCGATCTATGCAGA R: AGAGACAAAGCTTAAAGACCCA	(TTC)9	192-243	53
LG10	F: CTCGCCCTTCAAAGCCCTAA R: TTTTCGTTGTGGCTGCGAAG	(CT)9	166-186	56
LG11	F: CCGCTCGCAATGATGATTGG R: AGGCTTAGGCTTGTGCATGA	(TGTA)14	112-130	55
LG12	F: AGATCTCTCCCACTTCCCCA R: GCTACCCTGATGCCCAAAGT	(TGGA)8	142-148	54
LG13	F: ATGTGTGGTCTTGGCCCATC R: TGGTAGTAAAAGGCAGGGCG	(TTCTCC)5	156-174	55
LG14	F: CAAGACCCTTCCTCCACCAC R: ACCAAGATCGTCTCCCAGGT	(GGCGGT)6	160-178	55
LG15	F: CTTCGTCGACTCCCTCATCG R: GTTTCTTCCTGCAAACGCGT	(TA)9	146-164	56

**Table 6.** Genetic diversity statistics for two *L. discolor* populations based on 15 polymorphic EST-SSR primers.

**Locus**	**Dien Khanh (DK) Population** **(N = 24)**					**Khanh Son (KS) Population** **(N = 44)**					**Total**								
	**Na**	**Ne**	**PIC**	**Ho (SE)**	**He (SE)**	**Na**	**Ne**	**PIC**	**Ho (SE)**	**He (SE)**	**Na**	**Ne**	**PIC**	**Ho (SE)**	**He (SE)**	***Fis* (SE)**	***Fit* (SE)**	***Fst* (SE)**	**Nm (SE)**
LG01	3	2.58	0.75	0.92***	0.61	3	2.15	0.75	0.55***	0.53	3.00	2.36	0.86	0.57**	0.73	-0.28	-0.19	0.07	3.48
LG02	3	2.17	0.57	0.75	0.54	4	2.32	0.50	0.86	0.57	3.50	2.24	0.55	0.55	0.81	-0.46	-0.22	0.16	1.32
LG03	5	2.82	0.50	0.92	0.64	5	3.92	0.51	0.89***	0.75	5.00	3.37	0.61	0.70***	0.90	-0.30	-0.15	0.11	2.00
LG04	3	1.68	0.62	0.46	0.41	3	1.77	0.72	0.27^‡^	0.44	3.00	1.73	0.76	0.42^‡^	0.37	0.13	0.37	0.27	0.66
LG05	4	2.74	0.42	0.67	0.64	4	3.20	0.41	0.84	0.69	4.00	2.97	0.50	0.66***	0.75	-0.14	-0.11	0.02	10.75
LG06	4	2.95	0.61	0.29^‡^	0.66	4	3.32	0.65	0.91	0.70	4.00	3.13	0.64	0.68	0.60	0.12	0.14	0.02	10.92
LG07	2	1.88	0.64	0.75	0.47	3	2.35	0.66	0.77*	0.57	2.50	2.12	0.65	0.52	0.76	-0.46	-0.16	0.20	0.97
LG08	2	1.95	0.42	0.42	0.49	2	1.71	051	0.23*^‡^	0.42	2.00	1.83	0.60	0.45***^‡^	0.32	0.29	0.30	0.02	15.36
LG09	3	1.67	0.42	0.50	0.40	3	2.54	0.36	0.66	0.61	3.00	2.11	0.37	0.50	0.58	-0.15	0.07	0.19	1.08
LG10	2	1.38	0.41	0.33	0.28	3	1.60	0.56	0.45	0.37	2.50	1.49	0.58	0.33^‡^	0.39	-0.21	0.30	0.42	0.34
LG11	3	2.20	0.30	0.92	0.55	4	2.64	0.37	0.61	0.62	3.50	2.42	0.49	0.58	0.77	-0.31	-0.24	0.06	4.21
LG12	2	1.95	0.50	0.50**	0.49	2	1.95	0.59	0.48	0.49	2.00	1.95	0.59	0.49	0.49	0.00	0.02	0.03	9.17
LG13	3	2.03	0.42	0.71	0.51	3	2.02	0.40	0.68	0.51	3.00	2.03	0.40	0.51	0.70	-0.37	0.06	0.31	0.55
LG14	3	1.68	0.47	0.50	0.41	3	2.60	0.45	0.45	0.62	3.00	2.14	0.68	0.51	0.48	0.07	0.22	0.16	1.27
LG15	3	2.23	0.39	0.63	0.55	4	2.35	0.57	0.86	0.57	3.50	2.29	0.57	0.56***	0.74	-0.32	-0.02	0.23	0.83
Mean	3	2.13	0.50	0.62 (0.05)	0.51 (0.03)	3.33	2.43	0.53	0.63 (0.06)	0.56 (0.03)	3.17	2.28	0.59	0.63 (0.04)	0.54 (0.02)	-0.16 (0.06)	0.03 (0.03)	0.15 (0.05)	4.19 (0.12)
Note: N, number of individuals of each populations; Na, number of alleles per locus; Ne, number of effective alleles per locus; Ho, observed heterozygosity; He, expected heterozygosity; PIC, polymorphism information content; significant deviation from the Hardy-Weinberg equilibrium (* P<0.05, ** P<0.01, *** P<0.001); ^‡^significant possibility of the presence of null alleles detected by MICRO-CHECKER. Fis, inbreeding coefficient, Fit coefficient of total inbreeding, F_ST_, genetic differentiation index of Weir and Cockerham (1984); Nm, gene flow.																			

**Table 7.** Analysis of molecular variance from natural populations for *L. discolor* species produced

**Source of variation**	**df**	**Sum of squares**	**Variance components**	**Percentage** **variation (%)**	**Fixation indices**
Among populations	1	91.396	1.414	23	*Fis* = -0.141 *Fst* = 0.255*** Fit = 0.15**
Among individuals within populations	66	234.273	0.000	0	
Within individuals	68	320.500	4.713	77	
Total	135	646.169	6.127	100	
*Note*: df, degree of freedom; ^∗^P < 0.05, ^∗∗^P < 0.01, ***p < 0.001					
