# Supplementary material for: Transcriptome characterization and population genetics of Ludisia discolor (Ker Gawl.) A.Rich (Orchidaceae): implication for its conservation in Vietnam
Source: Biodivers Data J. 2026 Jan 8;14:e173579. doi: 10.3897/BDJ.14.e173579 (PMC12809156; doi:10.3897/BDJ.14.e173579)
Supplement: Supplementary material 1 — Gene Ontology [file bdj-14-e173579-s001.docx]

| **Table S2.** KOG functional annotation distribution of unigenes in transcriptome for L. discolor | | | |
| --- | --- | --- | --- |
| **Classification** | **Code** | **Code Function** | **Gene Count** |
| Cellular processes and signaling | D | Cell cycle control, cell division, chromosome partitioning | 334 |
|  | M | Cell wall/membrane/envelope biogenesis | 190 |
|  | N | Cell motility | 5 |
|  | O | Posttranslational modification, protein turnover, chaperones | 1344 |
|  | T | Signal transduction mechanisms | 1141 |
|  | U | Intracellular trafficking, secretion, and vesicular transport | 681 |
|  | V | Defense mechanisms | 116 |
|  | W | Extracellular structures | 24 |
|  | Y | Nuclear structure | 48 |
|  | Z | Cytoskeleton | 247 |
| Information storage and processing | A | RNA processing and modification | 753 |
|  | B | Chromatin structure and dynamics | 177 |
|  | J | Translation, ribosomal structure and biogenesis | 690 |
|  | K | Transcription | 802 |
|  | L | Replication, recombination and repair | 401 |
| Metabolism | C | Energy production and conversion | 492 |
|  | E | Amino acid transport and metabolism | 528 |
|  | F | Nucleotide transport and metabolism | 133 |
|  | G | Carbohydrate transport and metabolism | 731 |
|  | H | Coenzyme transport and metabolism | 178 |
|  | I | Lipid transport and metabolism | 526 |
|  | P | Inorganic ion transport and metabolism | 313 |
|  | Q | Secondary metabolites biosynthesis, transport and catabolism | 459 |
| Poorly characterized | R | General function prediction only | 2911 |
|  | S | Function unknown | 900 |
