# Supplementary material for: Transcriptome characterization and population genetics of Ludisia discolor (Ker Gawl.) A.Rich (Orchidaceae): implication for its conservation in Vietnam
Source: Biodivers Data J. 2026 Jan 8;14:e173579. doi: 10.3897/BDJ.14.e173579 (PMC12809156; doi:10.3897/BDJ.14.e173579)
Supplement: Supplementary material 3 — Summary of analyses [file bdj-14-e173579-s003.docx]

| **Table S4.** Summary of SSR types in the transcriptome of L. discolor | | | | | |
| --- | --- | --- | --- | --- | --- |
| **Repeat motif** | **Number** | **Percentage** | **Repeat motif** | **Number** | **Percentage (%)** |
| *Di-nucleotide* | | | *Tetra-nucleotide* | | |
| AC | 9 |  | AAAC | 2 |  |
| AG | 193 |  | AAAG | 1 |  |
| AT | 52 |  | AAAT | 13 |  |
| CA | 21 |  | AACA | 1 |  |
| CG | 5 |  | AAGC | 1 |  |
| CT | 214 |  | AATA | 2 |  |
| GA | 163 |  | AATC | 1 |  |
| GC | 5 |  | AATT | 1 |  |
| GT | 14 |  | AGAA | 2 |  |
| TA | 61 |  | AGCC | 1 |  |
| TC | 176 |  | ATAC | 1 |  |
| TG | 40 |  | ATGC | 1 |  |
| Total | **953** | **35.24** | ATTG | 1 |  |
| *Tri-nucleotide* | | | ATTT | 4 |  |
| AAC | 1 |  | CAAT | 1 |  |
| AAG | 90 |  | CTCA | 3 |  |
| AAT | 34 |  | CTCG | 1 |  |
| ACA | 3 |  | GACC | 2 |  |
| ACC | 9 |  | GCTG | 1 |  |
| AGA | 58 |  | GCGA | 1 |  |
| AGC | 27 |  | GATC | 1 |  |
| AGG | 7 |  | GTAT | 1 |  |
| AGT | 2 |  | GTTC | 1 |  |
| ATA | 9 |  | GGAG | 1 |  |
| ATC | 61 |  | GCTC | 1 |  |
| ATG | 25 |  | TAAA | 3 |  |
| ATT | 29 |  | TATG | 4 |  |
| CAA | 7 |  | TCTA | 1 |  |
| CAC | 9 |  | TCGC | 1 |  |
| CAG | 36 |  | TATT | 1 |  |
| CAT | 34 |  | TCTT | 2 |  |
| CCA | 13 |  | TGGA | 1 |  |
| CCG | 35 |  | TGTA | 2 |  |
| CCT | 23 |  | TTCT | 1 |  |
| CGA | 1 |  | TTTA | 4 |  |
| CGC | 42 |  | TTTC | 2 |  |
| CGG | 55 |  | Total | **68** | **2.51** |
| CTA | 4 |  | *Penta-nucleotide* | | |
| CTC | 24 |  | TGGAT | 2 |  |
| CTG | 18 |  | GTTTT | 3 |  |
| CTT | 81 |  | ATCCT | 1 |  |
| GAA | 106 |  | TTCTC | 1 |  |
| GAC | 7 |  | Total | **7** | **0.26** |
| GAG | 11 |  | *Hexa-nucleotide* | | |
| GAT | 35 |  | TTCTCC | 1 |  |
| GCA | 25 |  | GGCGGT | 2 |  |
| GCC | 52 |  | CGCCAC | 1 |  |
| GCG | 41 |  | TCTTCA | 1 |  |
| GCT | 29 |  | TGGAGA | 1 |  |
| GGA | 34 |  | CTCCTG | 1 |  |
| GGC | 73 |  | CTCCTG | 1 |  |
| GGT | 7 |  | GTGATG | 1 |  |
| GTA | 1 |  | TTCCAT | 1 |  |
| GTG | 5 |  | GGGGCA | 1 |  |
| GTT | 12 |  | CCACCG | 1 |  |
| TAA | 13 |  | CATGCC | 1 |  |
| TAC | 2 |  | Total | **13** | **0.48** |
| TAT | 26 |  | **Sum** | **2,704** |  |
| TCA | 44 |  |  |  |  |
| TCC | 16 |  |  |  |  |
| TCG | 3 |  |  |  |  |
| TCT | 129 |  |  |  |  |
| TGA | 102 |  |  |  |  |
| TGC | 36 |  |  |  |  |
| TGG | 10 |  |  |  |  |
| TTA | 16 |  |  |  |  |
| TTC | 72 |  |  |  |  |
| TTG | 19 |  |  |  |  |
| Total | **1663** | **61.50** |  |  |  |
